# STANDARDIZATION OF GERONTOLOGY MICRO-CREDENTIALS: IMPLICATIONS FOR THE FREE MARKET

**DOI:** 10.1093/geroni/igae098.1003

**Published:** 2024-12-31

**Authors:** Jennifer Ellis

**Affiliations:** Northwood Technical College, Superior, Wisconsin, United States

## Abstract

Micro-credentials are an emerging market for gerontology and thus require understanding before we can advance and identify their contribution to the field. In this presentation, we will review and discuss the standardization of gerontology micro-credentials in the United States. The function and role of micro-credentials vary beyond just disciplinary backgrounds and are also based on institution type and geographic location. The diversity of short-term credit- and non-credit micro-credentials expands in response to the workforce’s training needs. Yet, the assessment of certificates and other micro-credentials is in its infancy. There are limited directions, competing viewpoints, and misconceptions about what micro-credentials are and who they serve. The lack of infrastructure or expectations from the gerontology community has opened the free market to respond at a time when credit-based programs are waning. What are the implications of aligning industry standards with all short-term training and education, and how do they correlate with the needs of aging service providers? This presentation aims to open dialogue with educators, providers, and systems leaders to extrapolate the opportunities and challenges with gerontology-focused micro-credentials.

